# Evaluation of the Performances of the Rapid Test RESIST-5 O.O.K.N.V Used for the Detection of Carbapenemases-Producing Enterobacterales

**DOI:** 10.3390/antibiotics10080953

**Published:** 2021-08-06

**Authors:** Assiya El Kettani, Fakhreddine Maaloum, Nehemie Nzoyikorera, Mohamed Khalis, Khalid Katfy, Houria Belabbes, Khalid Zerouali

**Affiliations:** 1 Bacteriology-Virology and Hospital Hygiene Laboratory, University Hospital Centre Ibn Rochd, Casablanca 20503, Morocco; fakher.7@gmail.com (F.M.); nzoyikorera@yahoo.fr (N.N.); khalidkatfy@hotmail.com (K.K.); belhor2001@yahoo.fr (H.B.); khalid.zerouali2000@gmail.com (K.Z.); 2 Department of Microbiology, Faculty of Medicine and Pharmacy, Hassan II University, Casablanca 20503, Morocco; 3 International School of Public Health, Mohammed VI University of Health Sciences, Casablanca 82403, Morocco; mkhalis@um6ss.ma

**Keywords:** Enterobacterales, carbapenemase, rapid test

## Abstract

Background: The emergence of carbapenemase-producing Enterobacterales (CPE) is a public health problem, requiring rapid and reliable diagnostic methods. The aim is to compare the new rapid immunochromatographic (IC) test: RESIST-5 O.O.K.N.V with PCR and the predictive model of EUCAST algorithm for the detection of CPE. Methods: A longitudinal cross-sectional study was carried out in the bacteriology-virology laboratory of the Ibn Rochd-Casablanca University Hospital, from 1 February 2019 to 28 February 2020, concerning strains with reduced sensitivity to Ertapenem. The identification of bacterial species was carried out according to the standard criteria of microbiology and antibiogram according to CASFM-EUCAST 2019 recommendations. The sensitivity and specificity of the rapid IC test were calculated. Results: The results of the new IC test showed a sensitivity and specificity of 100% for the detection of OXA-48 and NDM. These carbapenemases were detected simultaneously with a sensitivity and specificity of 100%. OXA-48 was the most common carbapenemas found (36%), followed by NDM (24%) and (13.4%) cases of OXA-48 and NDM coexistence. Conclusion: The rapid IC test could be a rapid and effective diagnostic tool for detecting the most common carbapenemases in our context, and to accelerate the implementation of adequate antibiotic therapy and infection control measures in patients with CPE infections

## 1. Introduction

The emergence of carbapenemase-producing Enterobacterales (CPE) is a public health problem. In 2017, the World Health Organization ranked CPEs as a high priority due to their association with mortality and morbidity [1]. The resistance of Enterobacterales to carbapenems is mainly due to the production of enzymes, which hydrolyze the carbapenems that are part of beta lactamases. The plasmid genetic support of these resistances is at the origin of a transfer of resistance from one strain to another or even from one species to another [2]. Ambler’s classification categorizes beta lactamases into four groups (A, B, C, and D) according to their catalytic domain and their preferred substrate. Classes A, B, and D include, among others, the carbapenemases. Enzymes of class A: KPC, IMI, SME, and GES, as well as those of class D: OXA-48, OXA-181, OXA-23, OXA-40, and OXA-58 have serine at the catalytic site, while class B enzymes: NDM, VIM, IMP, and GIM are metallo-β-lactamases (MBL) with zinc at the active site [3,4]. There are currently 11 OXA-48 enzyme variants: OXA-48, OXA-48b, OXA-54, OXA-162, OXA-163, OXA-181, OXA-199, OXA-204, OXA-232, OXA-242, and OXA-247. These enzyme variants differ in some amino acid substitutions or deletions. They have been classified in the OXA-48-like group. They have reduced sensitivity to both carbapenems and broad-spectrum cephalosporins and are the most difficult resistance mechanism for clinical laboratories to detect, in particular, the OXA-163 variant. Indeed, although OXA-163 has lower carbapenemase activity than OXA-48, it shows increased activity towards extended spectrum cephalosporins which represents another challenge for identification [5]. Resistance to carbapenems can also, in rare cases, be attributed to mutations or other modifications that alter the affinity to penicillin-binding proteins. [6] Some Enterobacterales, such as Protea, have intrinsic resistance to imipenem and require resistance to other carbapenems to be classified as EPC.

The European Committee for Antimicrobial Susceptibility (EUCAST) defines thresholds for susceptibility to commercially available carbapenems annually, and has proposed a phenotypic algorithm for EPCs screening [7]. However, to confirm the production of carbapenemases and/or the presence of other resistance mechanisms, phenotypic biochemical and/or molecular genotypic tests should be performed.

Biochemical tests include Carba NP colorimetric tests and its derivative Blue Carba [8,9]. They are quick, easy to perform, and confirm phenotypically the carbapenemase production but no other resistance mechanisms, moreover, they are unable to identify the exact carbapenemase enzyme.

Immunochromatographic (IC) tests, such as the new RESIST-5 O.O.K.N.V. based on monoclonal antibodies generated by immunization of mice has been developed for rapid and easy identification of carbapenemases such as OXA-48, KPC, NDM VIM, and OXA-163 with a sensitivity of 97 to 100% and specificity of 100% [10].

However, the specific tests used to detect the presence of carbapenemas genes located on plasmids or mutations are normally based on amplification of potential genes present by PCR.

Whole genome sequencing allows the detection of carbapenemase genes as well as other mutations associated with resistance. However, this technology requires significant expertise and adequate equipment, which is not systematically available, and also a knowledge of the combined resistance mechanisms (e.g., mutations, levels of genes expression) [11].

Unlike in North America and some European countries, where KPC is the most predominant carbapenemase, in Southeast Asia and Latin America, NDM and other MBLs (e.g., IMP and VIM ) as well as OXA-48 are the most predominant carbapenemases [11]. In the Middle East, North Africa, and European countries such as Belgium and Spain, OXA-48 type enzymes are the most frequent carbapenemases among Enterobacteriaceae [12,13]. However, epidemiological data by region are still limited.

The objective of this study is to compare an immunochromatographic phenotypic method: the new RESIST-5 O.O.K.N.V. rapid test with a genotypic reference method (PCR) as well as with the predictive model of the EUCAST algorithm for the detection of EPCs, in order to use it in daily practice in the bacteriology-virology laboratory of the Ibn Rochd-Casablanca University Hospital.

## 2. Material and Methods

### 2.1. Study Design

This is a longitudinal cross-sectional study carried out in the bacteriology-virology and hospital hygiene laboratory of the Ibn Rochd-Casablanca University Hospital over a year (from 1 February 2019 to 28 February 2020).

### 2.2. Data Collection

It focused on Enterobacterales isolated from samples for diagnostic purposes and concerned strains with reduced sensitivity to ertapenem giving rise to suspicion of the production of carbapenemas according to the CASFM-EUCAST criteria. Duplicates were excluded.

The identification of the bacterial species was carried out according to standard microbiological criteria and the antibiogram according to the recommendations of CASFM-EUCAST 2019 [14]. The quality control strain ATCC *E.coli* 25922 was used. Isolates were subjected to the EPC screening algorithm, IC assays, and molecular assays for carbapenemase type determination. Any isolate meeting the criteria of the algorithm, but negative in the PCR test and in the IC test, was subjected to the CarbaNP colorimetric test to confirm the possible presence of the carbapenemase enzyme.

#### 2.2.1. The CASFM-EUCAST Algorithm (Modified)

In 2015, CASFM-EUCAST proposed a phenotypic algorithm for EPCS screening in strains not sensitive to carbapenems, it was updated in 2018.

All strains suspected of producing carbapenemas followed the algorithm with some modifications (ticarcillin/clavulanic acid, not tested for discs non-availability) [7].

#### 2.2.2. The Resist-5 O.O.K.N.V. K-Set (CORIS Bioconcept, Belgium)

Resist-5 O.O.K.N.V. is an immunochromatographic test for the detection and identification of carbapenemas. This test allows the rapid detection of one or more carbapenemas (OXA-48, NDM, VIM, KPC, and OXA-163) directly from a bacterial colony which grows on any culture medium [14,15].

#### 2.2.3. The RAPIDEC^®^ CARBA NP Test (Biomérieux, France)

The RAPIDEC^®^ CARBA NP Test is a colorimetric test which is based on the demonstration of acidification of the medium during hydrolysis of imipenem by a carbapenemase. The pH indicator changes its color (from red to yellow) when the medium becomes acidic, indicating the presence of a carbapenemase. This test is supposed to reflect any carbapenemase activity of an enterobacterium [16].

#### 2.2.4. PCR Tests for the Detection of Carbapenemas Genes

DNA extraction from bacterial strains was performed by heat shock. The molecular detection of the OXA-48, NDM, VIM, KPC, and IMP genes was carried out by multiplex PCR according to the protocol described by Dallene et al. [17].

### 2.3. Statistical Analysis

The specificity and sensitivity of the IC test as compared with the PCR test were calculated. The test results were compared with the predictive model of the EUCAST algorithm and the distribution of carbapenemases according to bacterial species was described.

Statistical analysis was performed by IBM SPSS Statistics v. 26 software.

## 3. Results

### 3.1. Description of Strains

During the study period, 97 strains of Enterobacterales isolated from diagnostic samples and with reduced sensitivity to ertapenem were identified in the bacteriology laboratory of the Ibn Rochd University Hospital. Among these strains, 93 isolates met the inclusion criteria for our study (four strains were duplicates).

**Figure 1:**
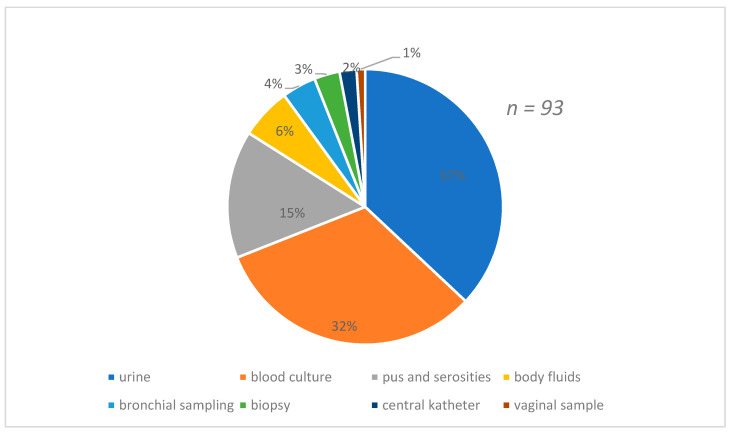
Distribution of the strains isolated according to the nature of the sample.

**Figure 2:**
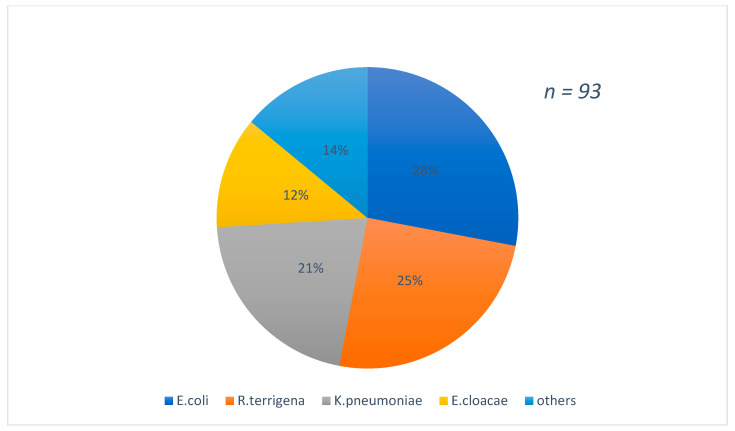
Distribution of strains isolated according to the species found.

Figure 1 shows the distribution of strains according to the types of samples which were predominant by urine (37%) and blood culture (32%) samples. The species found were predominantly *E.coli* 28%, followed by *R.terrigina* 25% and *K. pneumoniae* 21% Figure 2.

### 3.2. IC Assay Performance as Compared with PCR

The results of the IC test showed 100% agreement with the PCR results, with a sensitivity and specificity of 100%. They were also able to detect two carbapenemases simultaneously with a sensitivity of 100% and no cross-reaction was noted (Table 1).

The colorimetric test was applied to the 27 isolates suspected of EPC but negative by IC test and PCR. In 16/27 cases, the colorimetric test was positive.

### 3.3. Comparison of IC Test Results with the EUCAST Algorithm

**Figure 3:**
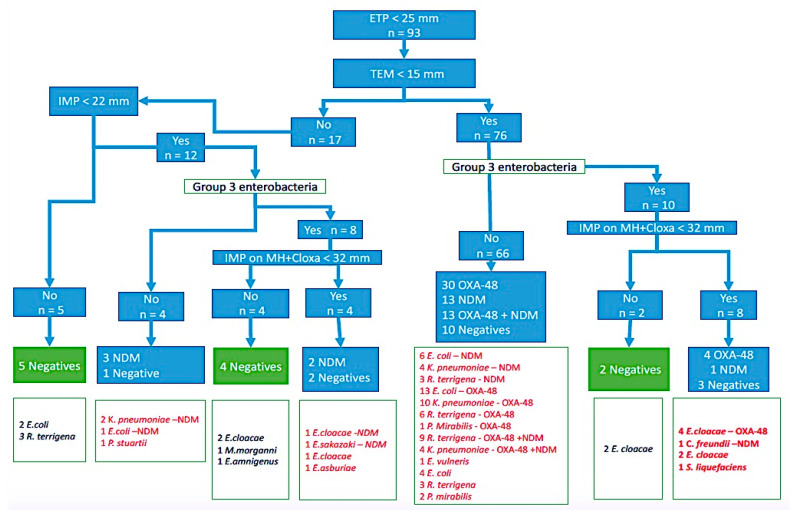
Comparison of IC test results with the EUCAST algorithm.

The EUCAST algorithm showed that 66/93 (70.9%) strains suspected to be EPC were confirmed by IC and PCR testing. Otherwise, all negative strains on the three tests were also negative by the algorithm (negative predictive value of 100%) Figure 3.

In the IC and PCR tests, OXA-48 was found the most in these strains, i.e., 34/93 (36%), followed by NDM 19/93 (24%), and 13/93 (13.4%) of cases OXA-48 and NDM coexistence, while 27/93 (29%) of cases were negative, 16 of which were positive in the colorimetric test.

**Figure 4:**
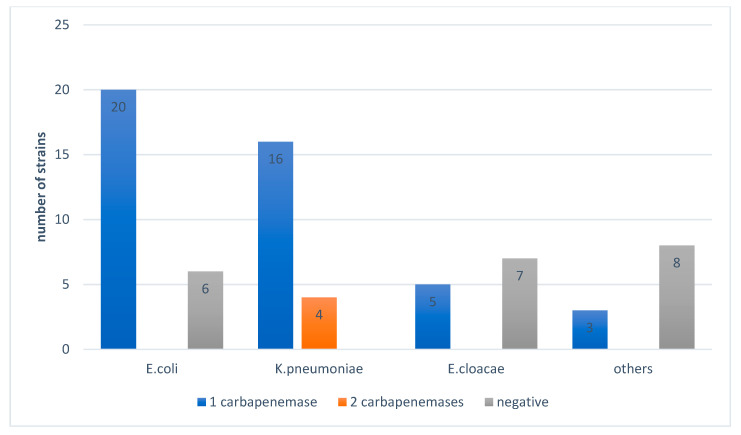
Distribution of the strains isolated according to the number of genes found.

Figure 4 shows the distribution of the strains isolated according to the number of genes found and shows the presence of coexistence of two carbapenemases (OXA-48 and NDM) in *K. pneumoniae* and *R. terrigena*. All strains of *K. pneumoniae* suspected of producing carbapenemas by the algorithm (10 strains) were confirmed with one or two enzymes.

**Figure 5:**
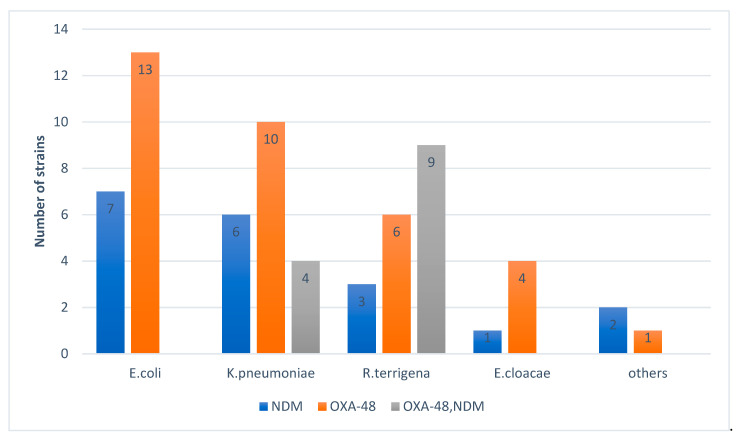
Distribution of carbapenemases according to the strains isolated.

Figure 5 shows a predominance of OXA-48 in *E. coli* and *K. pneumoniae* and a predominance of OXA-48 + NDM coexistence in *R. terrigena*.

In addition to this, all the strains producing carbapenemase type OXA-48 had a diameter of sensitivity to temocillin less than 15 mm (47 strains) and no case of coexistence of resistance genes was identified in group 3 Enterobacteria (10 strains).

## 4. Discussion

The results of the IC test showed 100% agreement with PCR for the detection of carbapenemas OXA-48 and NDM. They were also able to detect two carbapenemases simultaneously with a sensitivity and specificity of 100% and no cross reaction was obtained. The EUCAST algorithm showed an excellent negative predictive value of 100% on the strains tested. Our study showed a predominance of OXA-48, the emergence of the NDM strain, as well as cases of coexistence of carbapenemas (OXA-48 and NDM).

Our results agree with the results of Bianco et al. where the RESIST-5 O.O.K.N.V. detected OXA-48 and NDM-like carbapenemases from 4 h subcultures on blood culture samples with a sensitivity of 100% [18]. They also agree with the results of the study by Han et al. where the detection sensitivity of OXA-48 was 100%, however, the detection sensitivity of NDM was 97.1% [19]. Furthermore, studies carried out with the RESIST-4 O.K.N.V. (OXA-48, KPC, NDM, and VIM), RESIST-3 O.K.N. (OXA-48, KPC, and NDM), OXA-163/-48 K-Se T, and OXA-48 K-Se T based on the same principle as the RESIST-5 O.O.K.N.V., have also found a sensitivity of 100% for OXA-48 and a sensitivity for NDM ranging from 97.8% to 100% [20,21,22,23,24,25,26,27,28].

In the absence of OXA-163, VIM and KPC-producing Enterobacterales, we were unable to assess the performance of the RESIST-5 O.O.K.N.V. for their detection.

The IC RESIST-5 O.O.K.N.V test does not require specialized laboratories and sophisticated equipment for its performance, or extensive training such as that required for PCR. With a short handling time (less than 5 minutes), the test constitute a fast, reliable, inexpensive and easy to perform tool in all laboratories for the detection of EPC. However, they only detect the common carbapenemases, which leads to the non-detection of rare carbapenemases such as GES, IMI, and IMP. In our study, 16 strains were negative by the PCR and IC tests and positive by the Carba NP test, hence, genome sequencing is essential in order to elucidate the mechanisms of resistance of these strains to carbapenems. 

The Carba NP test has sensitivity ranging from 89% to 98% and specificity approaching 100%. The limitations of this test are some false negative results which were obtained with the OXA-48 and the interpretation of the results which may be subjective due to the slight color changes [29].

The IC test could be a rapid and reliable tool to speed up management, antibiotic therapy, and infection control measures in patients with infections caused by CPE. Indeed, rapid detection of carbapenemases can allow the prediction of multidrug-resistant phenotypes and rapid optimization of empirical antibiotic therapy based on local epidemiology. In fact, most Enterobacterales producing KPC or OXA-48 type carbapenemase are sensitive to ceftazidime-avibactam [30,31,32,33]. In contrast, ceftazidime-avibactam has no activity against metallo-beta lactamases (NDM, VIM, or IMP); therefore, other treatment options must be undertaken, such as tigecycline, colistin or other new antimicrobial agents (e.g., aztreonam-avibactam) [33,34,35,36,37,38].

## 5. Conclusions

The rapid IC test could be an efficient, rapid and practical diagnostic tool to detect the most common carbapenemases in Enterobacterales isolated at the CHU Ibn Rochd Casablanca. In addition, the implementation of antibiotic susceptibility databases and national, regional, and local surveillance studies at the hospital should be regular in order to help control the spread of CPEs.

## Figures and Tables

**Table 1:** IC assay performance as compared with PCR.

	PCR +	PCR −	Total
IC +	66	0	66
IC −	0	27	27
Total	66	27	93

## Data Availability

The data presented in this study are available on request from the corresponding author.
